# OVERVIEW AND EVIDENCE-BASE FOR PREVENTING LOSS OF INDEPENDENCE THROUGH EXERCISE (PLIÉ)

**DOI:** 10.1093/geroni/igac059.680

**Published:** 2022-12-20

**Authors:** Deborah Barnes, Margaret Chesney, Linda Chao, Wolf Mehling

**Affiliations:** University of California, San Francisco, San Francisco, California, United States; University of California, San Francisco, San Francisco, California, United States; University of California, San Francisco, San Francisco, California, United States; University of California, San Francisco, San Francisco, California, United States

## Abstract

PLIÉ was initially developed and tested in the adult day setting. Caregivers reported qualitative improvements in PLWD in psychological, physical, cognitive, and social domains that were supported by class video-recordings. Paired PLIÉ was adapted for the community setting and expanded to include dyads of PLWD and care partners (CPs) who participated in group classes together. PLWD reported significant improvements in self-rated quality of life, and CPs reported psychological, physical, cognitive, and social benefits for PLWD as well as themselves. We then adapted PLIÉ for people with mild cognitive impairment (MCI) and found that it significantly increased default mode network connectivity on resting-state functional MRI scans, while improving quantitative measures of cognitive function, social isolation, physical performance and self-regulation. We also implemented PLIÉ in a VA skilled nursing facility, where a post-implementation evaluation determined that residents, staff and family members all reported psychological, physical, cognitive and social benefits.

